# Farming non-typical sentient species: ethical framework requires passing a high bar

**DOI:** 10.1007/s10806-024-09928-y

**Published:** 2024-05-25

**Authors:** Siobhan Mullan, Selene S. C. Nogueira, Sérgio Nogueira-Filho, Adroaldo Zanella, Nicola Rooney, Suzanne D. E. Held, Michael Mendl

**Affiliations:** 1 School of Veterinary Medicine, University College Dublin, Belfield, Dublin 4, Ireland; 2 Departamento de Ciências Biológicas, Universidade Estadual de Santa Cruz, Rod. Jorge Amado, Km 16 - Salobrinho, Ilhéus, BA 45662-900 Brazil; 3 Departamento de Ciências Agrárias E Ambientais, Universidade Estadual de Santa Cruz, Rod. Jorge Amado, Km 16, Salobrinho, Ilhéus, BA 45662-900 Brazil; 4 Department of Preventive Veterinary Medicine and Animal Health, University of São Paulo, R. Duque de Caxias, 225, Caixa Postal 23, Pirassununga, SP 13635-900 Brazil; 5 Bristol Vet School, University of Bristol, Langford, Avon, BS40 5DU UK

**Keywords:** Livestock, Welfare, Farming, Wildlife, Neotropical animals

## Abstract

More widespread farming of species not typically used as livestock may be part of a sustainable approach for promoting human health and economic prosperity in a world with an increasing population; a current example is peccary farming in the Neotropics. Others have argued that species that are local to a region and which are usually not farmed should be considered for use as livestock. They may have a more desirable nutrient profile than species that are presently used as livestock. It may also reduce the pressure from hunting on other wild species, and cause less environmental damage than exotic species. We propose a sentiocentric utilitarian framework that could be used to decide whether species that are local, but generally not used as livestock, should be farmed. To illustrate the use of our decision-making framework, we employ two contrasting neotropical case studies: the Spotted Paca (Cuniculus paca) and the Capybara (Hydrochoerus hydrochaeris). We argue that it may be acceptable to use non-sentient species that are typically not farmed as livestock. However, research should determine whether farming them offers human, environmental or sustainability benefits. In addition, we recommend that if invertebrate species are considered for farming, research should be conducted to determine the likelihood that they are sentient. Finally, given the ethical failings of current livestock farming practices, we argue that a high bar must be met if ‘new’ species that are sentient are to be farmed.

## Introduction

Since the earliest domestication of our main terrestrial farmed animal species around 11,000–4,000 years ago (see MacHugh et al., 2017 for time frame and geography of domestication for key terrestrial vertebrate domestic species) our global livestock agricultural system has developed to produce large quantities of animal protein. In fact, current animal protein production is sufficient to provide the minimum protein requirements to the 2050 projected global population of 9.7 billion people (Berners-Lee et al., 2018). However, we are now more than ever aware that this has serious detrimental implications. Firstly, very large numbers of animals have suffered within confined systems. The main terrestrial farmed species–which include chickens, pigs, cattle and sheep—have species-specific welfare issues, perhaps most egregiously in the physically and behaviourally restrictive intensive farming systems developed over the last century for the majority of the 75bn broiler chickens, 7.8bn laying hens and 1.5bn pigsfarmed each year (FAOSTAT, 2022). For instance, global bovine dairy farming is characterised by separation of calves from the dam, and much animal production involves painful procedures such as castration, tail docking or disbudding. However, there has been a corollary of understanding of these species’ welfare needs and preferences, and in turn of those farming conditions that are conducive to good welfare—which include both traditional, non-intensive elements and also technological innovations. Secondly, the environmental costs of livestock farming have been well-established over the last two centuries and these costs continue to this day. Greenhouse gas emissions, loss of biodiversity and water pollution are amongst societal issues identified and their mitigation is receiving scientific attention (FAO 2018a, 2006). Thirdly, livestock farming has been implicated in increasing the risk to human public health, for example through zoonotic disease or by accelerating antimicrobial resistance (Bernstein & Dutkiewicz, 2021). Finally, despite the huge increase in livestock production, access to nutritious animal protein is poorly distributed to the global population. At the 2021 United Nations Food System Summit 42% of African 37% of Asian and 25% of Latin America/Caribbean national pathways to meet the UN Sustainable Development Goals by 2030 still included increased consumption of animal sourced foods (FAO, 2023).

The FAO define a sustainable food system as one “that is profitable throughout (economic sustainability)”, “has broad-based benefits for society (social sustainability)” and “has a positive or neutral impact on the natural environment (environmental sustainability)” (FAO 2018b). More widespread farming of non-typical animal species has been proposed as a solution to sustainably promote human health and economic prosperity in a world with an increasing human population (Smythe, 1991; Nogueira & Nogueira, 2011; Morais et al., 2022). It is suggested that non-typical species may be better suited to the local environment, climate and food (Cawthorn & Hoffmann, 2014; Hoffmann & Cawthorn, 2012), that farming local species may reduce the pressure on wild populations from hunting (Nogueira & Nogueira, 2004), and that some species may have a more desirable nutrient profile than the main typical farmed species (Saadoun & Cabrera, 2008). Additionally, there is evidence suggesting that some native species, such as the collared peccary (*Dicotyles tajacu*), a neotropical pig species, have higher productivity when farmed locally and cause less environmental damage than domesticated species such as cattle (Nogueira & Nogueira, 2011).

The term ‘non-typical’ over ‘novel’, ‘non-traditional’ or ‘non-conventional’ species for commercial farming has been used in this paper as some of the candidate species have a long history of being farmed on a small scale and/or in limited numbers (e.g. *Caviidae* sp.), whereas others have thus far not been farmed (e.g. *Octopoda* sp.). The suitability of both terrestrial and aquatic non-typical species have been investigated to varying degrees, including through specifically funded programmes (e.g. the €11.8 M DIVERSIFY project Mylonas et al., 2019). Proposed terrestrial non-typical species for significant farming expansion include crocodilians (*Crocodylus spp*.), ratites (*Struthio spp.*), camels (*Camelus dromedarius* and *Camelus bactrianus*), cane rats (*Thryonomys* spp.), giant rats (*Cricetomys gambianus*), and brush-tailed porcupines (*Atherurus africanus*) in Africa (Cawthorn & Hoffmann, 2014; Revol, 1995), paca (*Cunicullus paca*), capybara (*Hydrochoerus hydrochaeris*), peccaries (*Tayassu pecari; Dicotyles tajacu*), caimans (*Caiman yacare, Caiman crocodilius*) and river turtles (*Podocnemis expansa, Podocnemis unifilis*) in South America, kangaroos (*Osphranter and Macropus spp.*) in Australia, and a range of small lagomorphs and rodents across different continents (Cawthorn & Hoffmann, 2014). Aquatic non-typical species proposed for commercial farming include molluscs, echinoderms (Laguerre et al., 2020; Parisi et al., 2012), cephalopods (Estefanell et al., 2011; Vaz-Pires et al., 2004; Zheng et al., 2023), crustacean (The Fish Site, 2023) and numerous freshwater and marine fish (DIVERSIFY, 2018).

This paper examines the legitimacy of arguments to farm non-typical species by proposing a framework for deciding whether to commence trials to farm non-typical species and presents two case studies to illustrate the framework. Finally, recommendations for enabling practical implementation of our framework are proposed.

### Framework for Deciding Whether to Farm a Non-typical Species

**Fig. 1:**
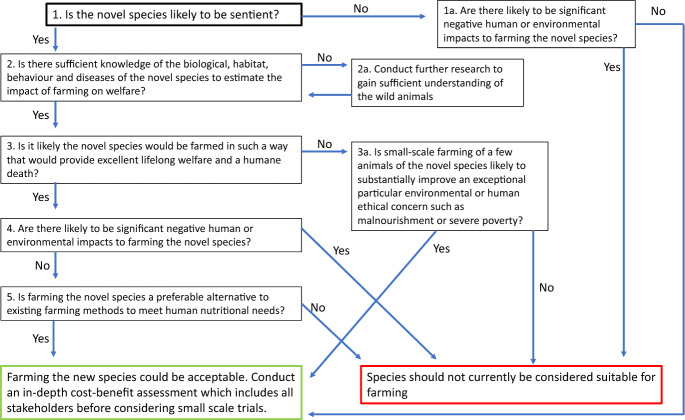
A decision-tree framework for determining ethical acceptability for farming non-typical species

We propose a decision-tree framework for determining the acceptability of farming non-typical species (see Fig. 1). It takes a utilitarian approach, with a clear sentiocentric leaning, to farming animals. In this, we regard animal experiences as being central to the concept of animal welfare and highly worthy of ethical consideration and aim to avoid, at almost all cost, significant or prolonged animal suffering. However, we accept that sometimes there are trade-offs with other sentient beings, in particular with humans, whose well-being has historically been entwined with consuming and farming animals, providing them with protein that promotes optimal health (FAO, WHO 2019) or economic prospects. In addition, there are many people who cannot access farmed animals through cost or accessibility and rely instead on hunting. In theory at least, most typically farmed animals can be provided with good lives throughout their whole natural lifespan under farming conditions. The framework therefore does not take a farming abolitionist approach, whilst recognising that many animals from typical farmed species suffer in existing systems. The consideration of laboratory-derived or plant-based alternative proteins is outside the scope of this framework. That environmental impacts can affect sentient human and non-human animals, be it through climate change, pollution or loss of habitat or biodiversity is recognised and therefore integrated within the decision-tree framework for farming non-typical species.

**Step 1: Is the species likely to be sentient?**

The framework is initially concerned with the likelihood of sentience- “the capacity, and level of awareness and cognitive ability, necessary to have feelings” (Broom, 2022). We consider the evidence for sentience to be extremely strong for all vertebrates and for the purposes of this framework take them to be sentient. Recent advances in cephalopod and decapod crustacean research have added weight to the likelihood of their sentience (Birch et al., 2021). For other species such as insects, molluscs, echinoderms and all other invertebrates there is limited evidence of sentience, whilst recognising that this also reflects a lack of scientific enquiry in general. A precautionary approach may be appropriate depending on the likelihood of sentience of species within this group. The potential concerns about farming any ‘likely non-sentient’ species therefore relate to impacts other than those on the individual animals, such as significant negative impacts on humans, other sentient species or the environmental impacts (including to the wild counterparts of the species). This is captured in

**Step 1a: Are there likely to be significant negative human or environmental impacts to farming the novel species?**

It is noted, for example, that there are concerns raised about the environmental impacts of farming insects for human or animal feed (Berggren et al., 2019; Jones, 2023; Tran et al., 2022) and that, although scatter-feeding whole larvae provides positive experiences for chickens even in intensive and barren environments (Ipema et al., 2020a, 2020b) if this practice were to further ‘prop-up’ intensive chicken farming, slowing change to more animal-welfare-friendly systems, this could be considered a significant negative impact on sentient animals and therefore should be avoided under the framework. Evaluation of the impact on a whole farming system is a ‘hard to estimate’ type of evidence but given the consequentialist nature of the framework, efforts should be made to ascertain all possible outcomes.

Relevant questions for this step include:

- Is the species a vertebrate, cephalopods or decapod crustacean?
- For all other species: how strong is the evidence of sentience? Is there any evidence of lack of sentience? What is the quality of that evidence?
- How does the likelihood of sentience affect any application of a precautionary principle for this species?

**Step 2: For sentient species: Is there sufficient knowledge of the biology, habitat, behaviour and diseases of the non-typical species to estimate the impact of farming on welfare?**

Here important questions are asked about the animal-centred platform on which it may be possible to build a case for farming non-typical species. With animals that are in the process of domestication their welfare will be highly influenced by their ability to cope with, and adapt to, a captive environment. A thorough understanding of their underlying biology and how it is likely to influence their welfare is essential (Zeuner, 1963). Further, studies can be used to systematically investigate the impact of biology, behavioural and ecological niche on ability to cope in captive environments (Mason, 2010), concluding, for example, that reproductive style (Lewis et al., 2022), home-range size (Clubb & Mason, 2003), cognitive capacity and foraging style (Mellor et al., 2021) are all predictors of captive welfare. In particular, basic knowledge about:

- Habitat, including range of different natural environments in which the animal is found
- Diet and manner of feeding
- Reproductive processes
- longevity
- Common diseases and mortality
- Social structures (including the need to be apart from conspecifics for solitary animals)
- Sensory abilities
- Cognitive abilities
- How they typically spend their time in the wild

are all essential to being able to make a priori estimations of whether captive environments are likely to be able to provide for even basic needs of an animal. For example, it has been shown that promiscuous ungulate species (those where both sexes have a high number of mating partners) had higher rates of performing repetitive non-functional behaviours (stereotypies) than polygynous species (where males mate with multiple females), and that males of polygamous (where females mate with multiple males) rather than monogamous (where a male and female pair for at least one breeding season) species had lower captive life expectancy (Lewis et al., 2022). In addition, understanding the sensory capabilities of the species is likely to give an insight into natural behavioural motivations and environments the animal has evolved for, and therefore help estimate the impact of captive conditions on welfare.

Further information about:

- Adaptability to different environments
- Reaction to humans and other predators

provides a fuller picture on which to base such a decision. In this step the proposed bar is not set very high. It should be possible to make relatively well-informed estimates of the impact of farming on welfare with the basic biological understanding. Where information is lacking, we propose to enter a loop of conducting further research and subsequent evaluation until sufficient evidence is available to progress.

Relevant questions for this step include:

- What evidence is available on key biological aspects of the species?
- What is the quality of this evidence? Where would it sit on a relevant hierarchy of evidence?
- Where is the evidence derived from (e.g. wild animals, which may be sparse, or captive animals which may have biases resulting from captivity)?
- Are there relevant biological aspects for which we have little, or poor quality, knowledge?
- How well would we be able to ‘fill in’ any biological gaps using information from closely related species?

**Step 3: Is it likely the non-typical species would be farmed in such a way that would provide excellent lifelong welfare and a humane death?**

This question addresses the likely practical implementation of farming of non-typical species. Few species thrive in restrictive environments and as we’ve seen with the typical farmed species this has increasingly been part of modern livestock farming systems. Here we need to first consider whether it’s possible that a non-typical species could, in theory at least, be farmed in such a way that good welfare at all life stages and death is promoted. Given that farming often relies on containing large numbers of animals in relatively small areas, there are particular challenges for solitary animals or those that live in small isolated territorial groups. In addition, animals with large home ranges, specialist diets or feeding methods may be hard to provide for and nocturnal animals bring challenges of providing adequate monitoring. Semi- wild or ranching style farming may afford high welfare opportunities for certain species (Nogueira et al., 2010), however these settings bring challenges of adequate monitoring and health care and ensuring any necessary interactions with humans, are humane despite little habituation. Secondly, for those species where high welfare farming is possible, we propose to consider the actual likelihood of this being employed. The economic and cultural settings will feature highly. If the farmers have little experience of high welfare farming and cannot sell their products above high welfare farming costs, then it’s unlikely to happen. Restrictive environments such as cages or small pens and/or barren environments cannot provide for good welfare, and many non-typical species face poor welfare at killing, at least initially, due to lack of dedicated systems capable of decreasing suffering during the process of killing the animals. In some instances, the species may be being farmed in small numbers already and following welfare evaluations, any existing good practices may serve as a guide for upscaling, at least in the short term.

Relevant questions for this step include:

- How likely is it that the biological needs can be easily met with regard to diet, environment, social structure and daily activity?
- How likely is it that positive welfare experiences could be integrated into the farming system?
- Is it likely we can humanely and safely kill the species?
- How likely is high welfare farming, considering any practical and economic constraints?
- Is this species farmed already? What is the welfare of those animals?
- For those species where welfare is likely to be compromised a supplementary question is asked:

**Step 3a: Is small-scale, temporary farming of a few animals of the non-typical species likely to substantially improve an exceptional environmental concern (such as recovery of an endangered species) or human ethical concern (such as malnourishment or severe poverty)?**

This step concludes that there could be occasions where an over-riding ethical priority could trump animal welfare. For example, there could be people suffering from severe malnutrition, or where local hunting was having a significant impact on biodiversity or endangered species, where small-scale farming of non-typical species in a low-welfare way may be justified. The argument here is prefaced with the recognition that even though farming a non-typical species in a low welfare way may appear initially to be acceptable, as with ‘Bateson’s cube’ for animals used for scientific research (Bateson, 1986) in many instances there could be other preferable mechanisms employed to tackle the issue. For example, Kuhnlein and Chotiboriboon (2022) discuss a range of policy options and education initiatives to strengthen the food systems of Indigenous Peoples based on a fundamental recognition of land rights and the importance of the whole food system to all generations.

Relevant questions for this step include:

- What is the exceptional environmental or human ethical concern that could trump animal welfare?
- Are there preferable alternative ways to resolve that concern without farming non-typical species in a low welfare way?
- How can the animal welfare impact be limited? For example, reducing the number of animals affected, employing a time limitation until preferable systems are in place.

For those species where life-long good welfare and a humane death are likely to be achievable any other potential negative impacts are considered:

**Step 4: Are there likely to be significant negative human or environmental impacts to farming the non-typical species?**

Taking a holistic OneWelfare approach considering “the interrelationships between animal welfare, human wellbeing and the physical and social environment” (Pinillos (ed) 2018) it is proposed to consider the impact of farming non-typical species on human welfare and the environment. Negative human impacts could include safety risks, for example from large or aggressive animals, as well as risks to human health through exposure to disease. Although often proposed as a solution to the negative environmental impact of typically farmed species, it is important to estimate as accurately as possible the environmental impacts of farming non-typical species. These could include local deforestation or pollution, impact of growing and/or transporting feed and the carbon footprint of the whole production cycle. Non-typical species have received little advanced genetic selection for productivity so far, and whilst this has led to physical and physiological conditions that result in very poor welfare of typical farmed species which should not be replicated in non-typical species, it has resulted in significant productivity gains. For example, over 48 years between 1956 and 2005 the standard broiler chicken increased growth rate by 400% and improved food conversion ratio by 50% (Zuidhof et al., 2014). Farming non-typical species may offer opportunities to harness local feedstuffs and avoid some of the existing farming problems, however we concede in this step that there are likely to be few species with no negative consequences so an evaluation of the degree of likely impact is required in order to decide whether it could be acceptable. We suggest that any essential negative aspects, for example greenhouse gas emissions inherent to gut fermenting species, that have the potential for mitigation are likely to be more acceptable.

Relevant questions for this step include:

- Are there any likely human safety or public health implications for farming the species?
- Are there aspects of farming that are likely to result in high greenhouse gas emissions, loss of biodiversity or pollution?
- Where does the feed for the animals come from? Can it be grown locally with low emissions?
- Can the species be farmed in harmony with the local environment and resources?
- Are there disease risk implications for local wild animals?
- Will the removal of native animals be required to set up farms have significant impacts on local ecosystems?

Finally, in the absence of expected significant negative consequences the framework asks:

**Step 5: Is farming the non-typical species a preferable alternative to existing food production methods to meet human nutritional requirements?**

Here, for the final step, it is suggested that beginning small scale trials of farming a non-typical species could be acceptable if the previous conditions are satisfied and farming the non-typical species has a better harm: benefit ratio to relevant comparator existing farmed species. In theory, it could potentially be acceptable if it has an equal harm: benefit ratio, but given we expect the level of uncertainty around estimates of human and animal welfare and environmental impacts to be higher for non-typical species than widely farmed species, a precautionary principle has been built in to account for any margin of error. In practice, given the current poor welfare and high environmental impact of so many animals of typical species, it is likely that, providing the previous steps are met, they will be at least equal to many of the widespread existing systems where feed travels long distances and pollution is poorly managed. When evaluating this final step, it is important to consider the local existing food systems, and whether nutritional animal protein needs are currently met. Where existing animal protein consumption is excessive to population need we conclude in this step that the existing food production comparator should be with the impact for the minimal need (see Willett et al., 2019 for reference values), notwithstanding that it would take some societal adaptation to achieve that. Where people are already engaging in small scale or extensive farming systems these may be more likely to be comparable to some of the non-typical farming systems proposed.

Relevant questions for this step:

- What is the impact of the relevant existing comparator farming systems for the specific human population? i.e. what species are currently eaten, and from what systems?
- Would the non-typical farming system deliver benefits to the local population over existing systems, particularly considering socio-economic, geographical or other limitations for accessing existing animal protein?
- Are there particular regions, areas or people who are particularly likely to benefit from farming non-typical species?

## Case Examples

We have chosen to illustrate use of the framework with two case examples of neotropical animals which are the particular expertise of co-authors SN, SN and AZ.

### Case example 1: Spotted Paca (Cuniculus paca)

**Step 1: Is the non-typical species likely to be sentient?**

Answer: Yes- the Spotted or Lowland Paca is a sentient mammal.

**Step 2: Is there sufficient knowledge of the biology, habitat, behaviour and diseases of the non-typical species to estimate the impact of farming on welfare?**

There has been a moderate amount of research on the behaviour of paca, who are thought to be nocturnally foraging neotropical rodents, weighing around 6–8 kg. They appear to be mostly solitary, although with vocal complexity compatible with being a social species (Lima et al., 2018). They have large home ranges that include water bodies (50–200 Ha) (Gutierrez et al., 2017; Harmsen et al., 2018) and occupy cavities to rest during the day (Figueroa-de-Leon et al., 2016). Paca consume a range of vegetation, including fruits from more than 20 tree species, and adapt their diet to the available species in their location (Martinez-Cecenas et al., 2018). Paca reach sexual maturity around 4 months of age and tend to give birth to a single mature offspring as early as 9 months old (El Bizri et al., 2019). There have been several studies on physical and physiological aspects of spotted paca (e.g. da Silva et al., 2020; Leal et al., 2017; Rabello et al., 2021) and one investigating the link between vocalisations and affective state (Lima et al., 2022).

Answer: Yes- whilst there is still much to learn about paca, a reasonable estimate of the impact of captivity on their welfare is possible.

**Step 3: Is it likely the non-typical species would be farmed in such a way that would provide excellent lifelong welfare and a humane death?**

Paca are currently farmed in small numbers. In Brazil there are probably up to 100 farmers (Le Pendu et al., 2011; Trajano & Carneiro, 2019) with up to 400 paca on each farm. They are most commonly kept in barren pens housing two females and one male, although more extensive naturalistic farms are possible (Lall et al., 2020). One, or sometimes two, young are born to each female annually which reaches slaughter weight of about 5 kg in around 8–12 months depending on the diet provided. The nutritional needs of farmed paca, including caecotrophy, have been explored (Aldrigui et al., 2018; Nogueira et al., 2016). Farmed paca have not become habituated to humans and show extreme predator avoidance responses when captured (personal observation SM). Although home-made stunning devices and slaughter facilities have been made on some farms (personal observation SM), regulations may require that paca are transported long distances, something typically associated with poor welfare in other species, to be killed in slaughterhouses designed for typical species.

Answer: No—despite the potential dietary adaptability of the paca the solitary and nocturnal nature of the species mean it is unlikely to be suited to most farming settings.

**Step 3a: Is small-scale farming of a few animals of the non-typical species likely to substantially improve an exceptional particular environmental or human ethical concern such as malnourishment or severe poverty?**

There is currently a complex demand for paca meat. Firstly, paca are regularly hunted by Indigenous Peoples (Gallina et al., 2012; Valsecchi et al., 2014) and have even been considered the most hunted neotropical mammal (Harmsen et al., 2018). Secondly, paca meat, along with other ‘wild’ meats has a particular cultural status amongst some of the wider South American population, with connotations harking back to times of subsistence hunting as well as a current exclusivity. Hence, at present, paca meat is a highly expensive delicacy, with individual paca selling for $500 and paca dishes fetching around $100 in exclusive city restaurants. This combination of high desirability with a high price tag appears to drive illegal hunting, and farming paca has been proposed as a mechanism to relieve hunting pressures. Although overall paca are not a threatened species there are areas where their populations are under particular pressure (Gutierrez et al., 2017).

Answer: Probably not—whilst mechanisms should be employed to protect wild paca, including populations protected for hunting by Indigenous Peoples, farming is unlikely to be the most effective route to achieving this. However, if Indigenous Peoples who rely on paca hunting are experiencing malnutrition, then, if farming was deemed culturally acceptable to them, it could be possible to farm paca and possibly to exploit the high market value and purchase more low value animal protein. To become economically viable, however, studies testing the availability and welfare impact on paca of locally available foodstuffs still need to be done. Other short-term nutritional support should be provided alongside habitat protection measures to increase the abundance of foodstuffs.

*Final evaluation:* Species should not currently be considered suitable for farming.

### Case example 2: Capybara (*Hydrochoerus hydrochaeris*)

**Step 1: Is the non-typical species likely to be sentient?**

Answer: Yes- capybara are sentient mammals.

**Step 2: Is there sufficient knowledge of the biology, habitat, behaviour and diseases of the non-typical species to estimate the impact of farming on welfare?**

Capybara have been the subject of a large body of research to understand their biology and habitat (Moreira et al., 2012), and have also been kept extensively in captivity in zoos around the world. Capybara are the largest living rodent, weighing 40–60 kg, and are often described as semi-aquatic. They are hindgut fermenters (Lall et al., 2018) and most active at dawn/dusk feeding on a wide range of plants including grasses (Desbiez et al., 2011), with flexible diets depending on availability and competition (Quintana, 2002) leading to successful adaptation to a range of urban and other human environments (Magioli et al., 2023). They have home ranges from 5 to 16 ha (Herrera & Macdonald, 1989) and typically live in mixed habitats with water, forest cover and open foraging areas (Alho & Rondon, 1987) in typical social groups of 6–16 males and females (Herrera et al., 2011), but up to 49 individuals (Alho & Rondon, 1987). Capybara can breed all year round, and can have one or two litters a year of around four offspring, which are frequently suckled by other females in the group (Alvarez & Kravetz, 2006; Nogueira et al., 2000).

Answer: Yes- there is knowledge about all the main aspects of capybara biology and habitat to make a reasonable estimate of the impact of captivity on their welfare.

**Step 3: Is it likely the non-typical species would be farmed in such a way that would provide excellent lifelong welfare and a humane death?**

Early attempts to farm capybara in the 1970s-80 s were characterised by intensive practices which ran counter to their natural biology and behaviour, resulting in poor growth and reproductive performance as well as infanticide and aggression (Nogueira et al., 1999; Nogueira et al., 2012). However, since that time more naturalistic and sometimes semi-wild (i.e. utilising small areas of forest) settings have been employed to greater productive success and improved welfare (Nogueira et al., 2012; Nogueira & Nogueira, 2012) which offers opportunities for improved food security of a healthy meat (Nogueira-Filho & Nogueira, 2018). Captive bred capybara show much reduced predator (human) avoidance reactions, compared to wild caught animals (Nogueira et al., 2004). It seems likely that capybara could be farmed in a high welfare way and where this is more profitable for the farmer this will continue, however, regulation may be required to ensure that it remains the norm as farming develops. Currently in Brazil there are no bespoke slaughter premises for capybara and small-scale trials would be required to find the best solutions to protect welfare at killing.

Answer: Yes- currently it is likely capybara could be farmed in a high welfare way and with sufficient input, humane slaughter facilities could become available.

**Step 4. Are there likely to be significant negative human or environmental impacts to farming the non-typical species?**

Capybara are relatively safe to work with given their size and temperament (Nogueira & Nogueira, 2012). Their meat is “very nutritious, having high concentrations of protein and polyunsaturated fatty acids (PUFA) … low in cholesterol, and saturated fatty acids” (Ali & Jones, 2020). Capybara can thrive on a wide range of vegetation found in Latin America and diets can be tailored to local availability. In 2011 there were 98 commercial capybara farms registered in Brazil (Le Pendu et al., 2011). Although environmental impacts of capybara farms have not been assessed to date, that they can be farmed within existing jungle, not requiring deforestation, and in fact could support afforestation, offers substantial environmental benefits over some other species such as cattle. Wild capybara have been shown to carry a range of potentially zoonotic pathogens (Chiacchio et al., 2014) although it is unknown how much of a risk these would be in a farmed context.

Answer: No- there are not likely to be significant negative human or environmental impacts, although this may depend on public health measures and the scale of production. Suitable evaluation and modelling should be conducted to predict impact.

**Step 5. Is farming the non-typical species a preferable alternative to existing farming methods to meet human nutritional needs?**

In Brazil there is a great disparity in availability of affordable food with 15% of the population suffering from hunger and 9% from severe food insecurity in 2022 (PENSSAN Network, 2022). Farming capybara could offer opportunities for some populations to improve access to nutritious food, especially those dwelling in or near natural forest habitats. Alternatively, farming capybara could provide a source of income. In addition, wild capybara have had large population growths in some areas due to lack of predation and expansion of urban areas and so management of capybara may be a viable solution to feed protein-deficient people in these areas (Marchini and Crawshaw Jr 2015; Abra et al., 2021; Ruiz-Tagle et al., 2021). Whilst it is unclear whether large scale high welfare capybara farming could provide a substantial amount of food, there could be distinct welfare and environmental benefits through farming a locally adapted animal over the cattle, pigs and chickens currently farmed in Brazil.

Answer- possibly yes. For some groups of people and in certain circumstances farming capybara could be preferable to existing species.

*Final evaluation*: Species could be considered suitable for farming, but modelling of the production potential and evaluation of any environmental impact as well as sociological and policy analysis is required before small scale pilot farms are developed/ upscaling occurs.

### Application of the Framework to Other Species

Given the great variety of species eaten by humans, it is perhaps unsurprising that the calls to farm non-typical species have ranged across the taxa and continents. One prominent example within Europe has been the proposed development of an octopus farm—a sentient and solitary, carnivorous species. The case against octopus farming has been made elsewhere in the literature on the grounds of animal welfare and environmental impacts (Jacquet et al., 2019) and some regions are pre-emptively considering legislation to ban octopus farming (Healey, 2023). However, aquaculture may yet see the largest interest in farming non-typical species. There is the potential to start or upscale farming of likely non-sentient species with low greenhouse gas emissions, including bivalves (Tamburini et al., 2022) or sea cucumbers (Laguerre et al., 2020), although these are not without environmental impacts such as introduction of exotic species (McKindsey et al., 2007). We suggest that this framework is applied to these species to provide clarity on the ethical acceptability in each case.

## Conclusion

Our framework makes it clear that non-sentient non-typical species are likely to be most acceptable to farm, and we recommend that research and innovation is focussed here for cases where there are additional human and environmental benefits. However, it is possible that these species may not fulfil the nutritional needs of humans, hence other strategies that are sustainable, safe and humane may still be required to optimise human nutrition. It is also questionable whether many species previously considered non-sentientare actually sentient, therefore, as determining sentience is a key component of this framework, we also recommend that research is directed to provide clarity on this, particularly for invertebrate species. Whilst we don’t want to stifle farming innovation, we are compelled to argue, based on current practices for many typical species, to protect animal welfare a high bar needs to be passed before considering farming any non-typical sentient species.

## References

[CR1] Abra FD, Huijser MP, Magioli M, Bovo AAA, de Barros KMPM (2021). An estimate of wild mammal roadkill in São Paulo state Brazil. Heliyon.

[CR2] Aldrigui LG, Nogueira SLG, Mendes A, Altino VS, Ortmann S, Nogueira SSD (2018). Effect of different feeding regimes on cecotrophy behavior and retention of solute and particle markers in the digestive tract of paca (*Cuniculus paca*). Comparative Biochemistry and Physiology a-Molecular & Integrative Physiology.

[CR3] Alho CJR, Rondon NL (1987). Habitats, population densities, and social structure of capybaras (*Hydrochaeris Hydrochaeris, Rodentia*) in the Pantanal Brazil. Revista Brasileira De Zoologia.

[CR4] Ali AJ, Jones KR (2020). Nutritive value and physical properties of neo-tropical rodent meat-with emphasis on the Capybara (*Hydrochoerus hydrochaeris*). Animals.

[CR5] Alvarez MR, Kravetz FO (2006). Reproductive performance of capybaras (*Hydrochoerus hydrochaeris*) in captivity under different management systems in Argentina. Animal Research.

[CR6] Bateson P (1986). When to experiment on animals. New Scientist.

[CR7] Bernstein J, Dutkiewicz J (2021). A public health ethics case for mitigating zoonotic disease risk in food production. Food Ethics.

[CR8] Berggren A, Jansson A, Low M (2019). Approaching ecological sustainability in the emerging insects-as-food industry. Trends in Ecology & Evolution.

[CR9] Berners-Lee M, Kennelly C, Watson R, Hewitt CN (2018). Current global food production is sufficient to meet human nutritional needs in 2050 provided there is radical societal adaptation. Elementa-Science of the Anthropocene.

[CR10] Birch, J., Burn, C., Schnell, A., Browning, H., & Crump, A. (2021). Review of the Evidence of Sentience in Cephalopod Molluscs and Decapod Crustaceans. London, UK. 10.1002/brv.70125 41558838

[CR11] Broom DM (2022). Concepts and interrelationships of awareness, consciousness, sentience, and welfare. Journal of Consciousness Studies.

[CR12] Cawthorn D-M, Hoffmann LC (2014). The role of traditional and non-traditional meat animals in feeding a growing and evolving world. Animal Frontiers.

[CR13] Chiacchio RG, Prioste FE, Vanstreels RE, Knöbl T, Kolber M, Miyashiro SI, Matushima ER (2014). Health evaluation and survey of zoonotic pathogens in free-ranging capybaras (Hydrochoerus hydrochaeris). Journal of Wildlife Diseases.

[CR14] Clubb R, Mason G (2003). Captivity effects on wide-ranging carnivores. Nature.

[CR15] da Silva GP, Monteiro FOB, Pereira THD, de Matos SER, de Andrade RD, El Bizri HR (2020). Fetal bone development in the lowland paca (*Cuniculus paca, Rodentia, Cuniculidae*) determined using ultrasonography. Journal of Anatomy.

[CR16] Desbiez ALJ, Santos SA, Alvarez JM, Tomas WM (2011). Forage use in domestic cattle (*Bos indicus*), capybara (*Hydrochoerus hydrochaeris*) and pampas deer (*Ozotoceros bezoarticus*) in a seasonal Neotropical wetland. Mammalian Biology.

[CR17] DIVERSIFY (2018). Exploring the biological and socio-economic potential of new/emerging candidate fish species for the expansion of the European aquaculture industry.

[CR18] El Bizri HR, Fa JE, Valsecchi J, Bodmer R, Mayor P (2019). Age at sexual maturity, first parturition and reproductive senescence in wild lowland pacas (*Cuniculus paca*): Implications for harvest sustainability. Animal Reproduction Science.

[CR19] Estefanell J, Socorro J, Tuya F, Izquierdo M, Roo J (2011). Growth, protein retention and biochemical composition in Octopus vulgaris fed on different diets based on crustaceans and aquaculture by-products. Aquaculture.

[CR20] FAO (2006). Livestock's Long Shadow. Rome, Italy.FAO (2018a). World Livestock: Transforming the livestock sector through the Sustainable Development Goals. Rome, Italy

[CR21] FAO (2018). Sustainable food systems.

[CR22] FAO (2023). Contribution of terrestrial animal source food to healthy diets for improved nutrition and health outcomes–An evidence and policy overview on the state of knowledge and gaps.

[CR23] FAO, Who,  (2019). Sustainable healthy diets—Guiding principles.

[CR24] FAOSTAT (2022). Crops and Livestock Products. https://www.fao.org/faostat/en/#data/QCL Accessed 12th March 2024.

[CR25] Figueroa-de-Leon A, Naranjo EJ, Perales H, Santos-Moreno A, Lorenzo C (2016). Cavity occupancy by lowland paca (*Cuniculus paca*) in the Lacandon Rainforest, Chiapas Mexico. Tropical Conservation Science.

[CR26] Gallina S, Perez-Torres J, Guzman-Aguirre CC (2012). Use of the paca, Cuniculus paca (*Rodentia: Agoutidae*) in the Sierra de Tabasco State Park Mexico. Revista De Biologia Tropical.

[CR27] Gutierrez SM, Harmsen BJ, Doncaster CP, Kay E, Foster RJ (2017). Ranging behavior and habitat selection of pacas (*Cuniculus paca*) in central Belize. Journal of Mammalogy.

[CR28] Harmsen BJ, Wooldridge RL, Gutierrez SM, Doncaster CP, Foster RJ (2018). Spatial and temporal interactions of free-ranging pacas (*Cuniculus paca*). Mammal Research.

[CR29] Healey, P. (2023). Washington bill seeks a ban on octopus farms. https://www.speciesunite.com/news-stories/washington-bill-seeks-a-ban-on-octopus-farms. Accessed 4th July 2023.

[CR30] Herrera EA, Macdonald DW (1989). Resource utilization and territoriality in group-living capybaras (*Hydrochoerus hydrochaeris*). Journal of Animal Ecology.

[CR31] Herrera EA, Salas V, Congdon ER, Corriale MJ, Tang-Martinez Z (2011). Capybara social structure and dispersal patterns: Variations on a theme. Journal of Mammalogy.

[CR32] Hoffmann LC, Cawthorn D-M (2012). What is the role and contribution of meat from wildlife in providing high quality protein for consumption?. Animal Frontiers.

[CR33] Ipema AF, Bokkers EAM, Gerrits WJJ, Kemp B, Bolhuis JE (2020). Long-term access to live black soldier fly larvae (*Hermetia illucens*) stimulates activity and reduces fearfulness of broilers, without affecting health. Scientific Reports.

[CR34] Ipema AF, Gerrits WJJ, Bokkers EAM, Kemp B, Bolhuis JE (2020). Provisioning of live black soldier fly larvae (*Hermetia illucens*) benefits broiler activity and leg health in a frequency- and dose-dependent manner. Applied Animal Behaviour Science.

[CR35] Jacquet, J., Franks, B., Godfrey-Smith, P., & Sanchez-Suarez, W. (2019). The case against octopus farming. *Issues in Science and Technology, 35*(2), 37-+.

[CR36] Jones N (2023). Fungi bacon and insect burgers: A guide to the proteins of the future. Nature.

[CR37] Kuhnlein HV, Chotiboriboon S (2022). Why and how to strengthen indigenous peoples' food systems with examples from two unique indigenous communities. Frontiers in Sustainable Food Systems.

[CR38] Laguerre H, Raymond G, Plan P, Ameziane N, Bailly X, Le Chevalier P (2020). First description of embryonic and larval development, juvenile growth of the black sea-cucumber Holothuria forskali (*Echinodermata: Holothuroidea*), a new species for aquaculture in the north-eastern Atlantic. Aquaculture.

[CR39] Lall KR, Jones KR, Garcia GW (2018). Nutrition of six selected neo-tropical mammals in trinidad and tobago with the potential for domestication. Veterinary Sciences.

[CR40] Lall KR, Jones KR, Garcia GW (2020). Natural habitat, housing, and restraint of six selected neotropical animals in trinidad and tobago with the potential for domestication. Scientifica.

[CR41] Le Pendu Y, Guimaraes DA, Linhares A (2011). Estado da arte sobre a criação comercial da fauna silvestre brasileira. Revista Brasileira De Zootecnia.

[CR42] Leal LM, Samidi S, de Oliveira FS, Sasahara THC, Minto BW, Machado MRF (2017). Origin and distribution of the main arteries of the thoracic limb of *Cuniculus paca* (Linnaeus, 1766). Pesquisa Veterinaria Brasileira.

[CR43] Lewis K, Parker MO, Proops L, McBride SD (2022). Risk factors for stereotypic behaviour in captive ungulates. Proceedings of the Royal Society B-Biological Sciences.

[CR44] Lima AF, Lima SGC, Nogueira SLG, Held S, Paul E, Mendl M (2022). Vocal expression of emotions in farmed spotted paca (*Cuniculus paca*). Applied Animal Behaviour Science.

[CR45] Lima SGC, Sousa-Lima RS, Tokumaru RS, Nogueira SLG, Nogueira SSC (2018). Vocal complexity and sociality in spotted paca (*Cuniculus paca*). Plos One.

[CR46] MacHugh, D. E., Larson, G., & Orlando, L. (2017). Taming the Past: Ancient DNA and the Study of Animal Domestication. In H. A. Lewin, & R. M. Roberts (Eds.), *Annual Review of Animal Biosciences, * (Vol. 5, pp. 329–351, Annual Review of Animal Biosciences). 10.1146/annurev-animal-022516-022747 27813680

[CR47] Magioli M, Luz HR, Costa FB, Benatti HR, Piovezan U, Nunes FBP (2023). Plasticity in resource use explains the persistence of the largest living rodent in anthropized environments. Journal of Zoology.

[CR48] Marchini S, Crawshaw PG (2015). Human–wildlife conflicts in Brazil: A fast-growing issue. Human Dimensions of Wildlife.

[CR49] Martinez-Cecenas Y, Naranjo EJ, Henaut Y, Carrillo-Reyes A (2018). Foraging ecology of lowland paca (*Cuniculus paca*) in preserved and transformed areas of the Lacandon rainforest, Chiapas Mexico. Revista Mexicana De Biodiversidad.

[CR50] Mason GJ (2010). Species differences in responses to captivity: Stress, welfare and the comparative method. Trends in Ecology & Evolution.

[CR51] McKindsey CW, Landry T, O'Beirn FX, Davies IN (2007). Bivalve aquaculture and exotic species: A review of ecological considerations and management issues. Journal of Shellfish Research.

[CR52] Mellor EL, Kinkaid HKM, Mendl MT, Cuthill IC, van Zeeland YRA, Mason GJ (2021). Nature calls: Intelligence and natural foraging style predict poor welfare in captive parrots. Proceedings of the Royal Society B-Biological Sciences.

[CR53] Morais BHD, Cardoso DD, Costa JD, Mayor P, de Albuquerque NI, Chiste RC (2022). Use of wildlife as an alternative protein source: Collared peccary meat. Meat Science.

[CR54] Moreira JR, Ferraz KMP, Herrera EA, Macdonald DW (2012). Capybara: Biology, use and conservation of an exceptional neotropical species.

[CR55] Mylonas CC, Robles R, Tacken G, Banovic M, Krystallis A, Guerrero L (2019). New species for EU aquaculture. Food Science and Technology.

[CR56] Nogueira-Filho SLG, Nogueira SS (2018). Capybara meat: An extraordinary resource for food security in South America. Meat Science.

[CR57] Nogueira SLG, Bastos ID, Mendes A, Nogueira SSD (2016). Protein requirements of finishing paca (*Cuniculus paca*). Tropical Animal Health and Production.

[CR58] Nogueira SLG, Nogueira SSC, Silvius K, Bodner R, Fragoso J (2004). Captive breeding programs as an alternative for wildlife conservation in Brazil. People in Nature: Wildlife Manegement and Conservation in Latin America.

[CR59] Nogueira SLG, Pinheiro MS, Nogueira SS, Moreira J, Ferraz K, Herrera E, Macdonald D (2012). Confined and semi-confined production systems for Capybaras. Capybara.

[CR60] Nogueira SS, Nogueira-Filho SLG, Otta E, dos Santos Dias CT, de Carvalho A (1999). Determination of the causes of infanticide in capybara (*Hydrochaeris hydrochaeris*) groups in captivity. Applied Animal Behavioural Science.

[CR61] Nogueira SSC, Bernardi LG, Nogueira SLG (2004). A note on comparative enclosure facility usage by wild and captive-born capybaras (*Hydrochoerus hydrochaeris*). Applied Animal Behaviour Science.

[CR62] Nogueira SSC, Nogueira SLG (2011). Wildlife farming: An alternative to unsustainable hunting and deforestation in Neotropical forests?. Biodiversity and Conservation.

[CR63] Nogueira SSC, Nogueira SLG (2012). Capybara (*Hydrochoerus hydrochaeris*) behaviour and welfare: Implications for successful farming practices. Animal Welfare.

[CR64] Nogueira SSD, Silva MG, Dias CTD, Pompeia S, Cetra M, Nogueira SLG (2010). Social behaviour of collared peccaries (*Pecari tajacu*) under three space allowances. Animal Welfare.

[CR65] Nogueira SSDC, Otta EMMA, Dias CDS, Nogueira-Filho SLG (2000). Alloparental behavior in the capybara (*Hydrochoerus hydrochaeris*). Revista de Etologia.

[CR66] Parisi G, Centoducati G, Gasco L, Gatta PP, Moretti VM, Piccolo G (2012). Molluscs and echinoderms aquaculture: Biological aspects, current status, technical progress and future perspectives for the most promising species in Italy. Italian Journal of Animal Science.

[CR67] PENSSAN Network (2022). Food Insecurity and COVID-19 in Brazil. Brazil.

[CR68] Pinillos (ed), R. G. (2018). *One welfare: A framework to improve animal welfare and human well-being*: Cabi.

[CR69] Quintana RD (2002). Influence of livestock grazing on the capybara's trophic niche and forage preferences. Acta Theriologica.

[CR70] Rabello VC, Abdala FCM, Lebre EA, Gomes SP, Leal LM, de Oliveira FS (2021). The macro and micro-structure of the celiac and cranial mesenteric Ganglia in a long-lived rodent - Paca (*Cuniculus paca*, Linnaeus 1766). International Journal of Morphology.

[CR71] Revol B (1995). Crocodile farming and conservation, the example of Zimbabwe. Biodiversity & Conservation.

[CR72] Ruiz-Tagle NM, Nogueira-Filho SLG, Knowles TG, da Cunha S, Nogueira S (2021). Using predator feces as a repellent for free-ranging urban capybaras (Hydrochoerus hydrochaeris). Acta Ethologica.

[CR73] Saadoun A, Cabrera MC (2008). A review of the mineral content and technological parameters of indigenous sources of meat in South America. Meat Science.

[CR74] Smythe, N. (1991). Neotropical Wildlife Use and Conservation. In J. G. Robinson, & R. K.H. (Eds.). Chicago: University of Chicago Press.

[CR75] Tamburini E, Turolla E, Lanzoni M, Moore D, Castaldelli G (2022). Manila clam and mediterranean mussel aquaculture is sustainable and a net carbon sink. Science of the Total Environment.

[CR76] The Fish Site (2023). King crabs shows promise for aquaculture. https://thefishsite.com/articles/king-crabs-shows-promise-for-aquaculture. Accessed 2nd July 2023.

[CR77] Trajano, M. C., & Carneiro, L. P. (2019). Diagnóstico da Criação Comercial de Animais Silvestres no Brasil. Brazil.

[CR78] Tran HQ, Doan HV, Stejskal V (2022). Environmental consequences of using insect meal as an ingredient in aquafeeds: A systematic view. Reviews in Aquaculture.

[CR79] Valsecchi J, El Bizri HR, Figueira JEC (2014). Subsistence hunting of *Cuniculus paca* in the middle of the Solimoes River, Amazonas. Brazil. Brazilian Journal of Biology.

[CR80] Vaz-Pires P, Seixas P, Barbosa A (2004). Aquaculture potential of the common octopus (*Octopus vulgaris* Cuvier, 1797): A review. Aquaculture.

[CR81] Willett W, Rockstrom J, Loken B, Springmann M, Lang T, Vermeulen S (2019). Food in the anthropocene: The EAT-lancet commission on healthy diets from sustainable food systems. Lancet.

[CR82] Zeuner FE (1963). A History of Domesticated Animals.

[CR83] Zheng J, Qian YS, Zheng XD (2023). Effects of stocking density on juvenile Amphioctopus fangsiao (*Mollusca: Cephalopodasca: Cephalopoda*): Survival, growth, behavior, stress tolerance and biochemical response. Aquaculture.

[CR84] Zuidhof MJ, Schneider BL, Carney VL, Korver DR, Robinson FE (2014). Growth, efficiency, and yield of commercial broilers from 1957, 1978, and 2005. Poultry Science.

